# Network Pharmacology to Unveil the Biological Basis of Health-Strengthening Herbal Medicine in Cancer Treatment

**DOI:** 10.3390/cancers10110461

**Published:** 2018-11-21

**Authors:** Jiahui Zheng, Min Wu, Haiyan Wang, Shasha Li, Xin Wang, Yan Li, Dong Wang, Shao Li

**Affiliations:** 1 MOE Key Laboratory of Bioinformatics; Bioinformatics Division Biology/Center for TCM-X, BNRist, TFIDT/Department of Automation, Tsinghua University, 100084 Beijing, China; zjh1228@yeah.net (J.Z.); minwu8@foxmail.com (M.W.); roymaleo@163.com (X.W.); 2 Department of Basic Medicine, School of Medicine, Tsinghua University, 100084 Beijing, China; wanghaiyan0917@sina.com (H.W.); ss-li14@tsinghua.org.cn (S.L.); dwang@biomed.tsinghua.edu.cn (D.W.); 3 State Key Laboratory of Bioactive Substances and Functions of Nature Medicines, Institute of Materia Medica, Chinese Academy of Medical Sciences & Peking Union Medical College, 100730 Beijing, China; lyhzytt@163.com

**Keywords:** traditional Chinese medicine, health strengthening herb, cancer treatment, network pharmacology, network target, high-throughput analysis

## Abstract

Health-strengthening (*Fu-Zheng*) herbs is a representative type of traditional Chinese medicine (TCM) widely used for cancer treatment in China, which is in contrast to pathogen eliminating (*Qu-Xie*) herbs. However, the commonness in the biological basis of health-strengthening herbs remains to be holistically elucidated. In this study, an innovative high-throughput research strategy integrating computational and experimental methods of network pharmacology was proposed, and 22 health-strengthening herbs were selected for the investigation. Additionally, 25 pathogen-eliminating herbs were included for comparison. First, based on network-based, large-scale target prediction, we analyzed the target profiles of 1446 TCM compounds. Next, the actions of 166 compounds on 420 antitumor or immune-related genes were measured using a unique high-throughput screening strategy by high-throughput sequencing, referred to as HTS^2^. Furthermore, the structural information and the antitumor activity of the compounds in health-strengthening and pathogen-eliminating herbs were compared. Using network pharmacology analysis, we discovered that: (1) Functionally, the predicted targets of compounds from health strengthening herbs were enriched in both immune-related and antitumor pathways, similar to those of pathogen eliminating herbs. As a case study, galloylpaeoniflorin, a compound in a health strengthening herb *Radix Paeoniae Alba* (*Bai Shao*), was found to exert antitumor effects both in vivo and in vitro. Yet the inhibitory effects of the compounds from pathogen eliminating herbs on tumor cells proliferation as a whole were significantly stronger than those in health-strengthening herbs (*p* < 0.001). Moreover, the percentage of assay compounds in health-strengthening herbs with the predicted targets enriched in the immune-related pathways (e.g., natural killer cell mediated cytotoxicity and antigen processing and presentation) were significantly higher than that in pathogen-eliminating herbs (*p* < 0.05). This finding was supported by the immune-enhancing effects of a group of compounds from health-strengthening herbs indicated by differentially expressed genes in the HTS^2^ results. (2) Compounds in the same herb may exhibit the same or distinguished mechanisms in cancer treatment, which was demonstrated as the compounds influence pathway gene expressions in the same or opposite directions. For example, acetyl ursolic acid and specnuezhenide in a health-strengthening herb *Fructus Ligustri lucidi* (*Nv Zhen Zi*) both upregulated gene expressions in T cell receptor signaling pathway. Together, this study suggested greater potentials in tumor immune microenvironment regulation and tumor prevention than in direct killing tumor cells of health-strengthening herbs generally, and provided a systematic strategy for unveiling the commonness in the biological basis of health-strengthening herbs in cancer treatment.

## 1. Introduction

China has a long history of using traditional Chinese medicine (TCM) for treating cancer [1]. A large amount of medication experience and clinical cases have been accumulated by TCM practitioners, which makes TCM contribute greatly to the development of China’s national health status. According to an urban basic medical insurance survey of inpatient use of health services in China from 2008 to 2010, 42.4% of oncology patients have used antineoplastic TCMs in the Chinese national medical insurance catalogue [2]. With increasing scientific evidence in biological, chemical, and medical research, as well as clinical trials, the use of traditional Chinese medicine in cancer treatment is gradually being recognized as a complementary and alternative therapy all over the world [3,4,5,6].

Traditionally, TCM adopts a relative and holistic point of view in cancer treatment. The clinical treatment strategy by strengthening health reflects the characteristics of focusing on regulatory effects instead of the antagonistic effects of TCM, and embodies the classical therapeutic theory that “pathogenic-qi cannot invade the body if health-qi remains strong” in the Canon of Internal Medicine (*Huangdi Neijing*). Therefore, an in-depth exploration on the antitumor effects exerted by health- strengthening herbs is meaningful and urgently needed. TCMs in the Chinese national medical insurance catalogue (2017) for oncology treatment are officially divided into two categories, including antitumor TCM and adjuvant TCM for tumors, which contain 40 TCM prescriptions in total [7]. Additionally, some health-strengthening prescriptions are widely applied in cancer treatment, such as *Sijunzi* decoction in colorectal cancer [8], *Shenqi Fuzheng* injection in colorectal cancer and breast cancer [9,10], *Shenling Baizhu San* in gastric cancer [11], and *Buzhong Yiqi* decoction in colorectal and lung cancer [12,13]. The wide application and the distinctive therapeutic strategy of health-strengthening herbs have given rise to the growing research interests in the investigation on the effects and the underlying mechanisms of health-strengthening herbs in cancer treatment. A large amount of research effort has been put into the studies on the biological basis of health-strengthening TCM from a variety of perspectives, such as their immune and metabolic regulatory effects. For example, previous studies on various health strengthening formulae (e.g., *Shenqi Fuzheng* injection, *Danggui Buxue* decoction, *Huangqi Jianzhong* decoction, and *Liu-wei-di-huang* pill) revealed their protective effect on immune functions in cancer therapy [14,15,16,17]. Metabolic regulatory function is also involved in the antitumor effects of health-strengthening formulae, suggested by the pharmacological studies on the *Liu-wei-di-huang* pill, *Jianpi Yiqi* decoction, and *Yishen Gukang* decoction [17,18,19]. Considering the situation that more studies emphasize the immune regulatory effects of health strengthening herbs, in this study, we took the immunological effects of health strengthening herbs as an example to explore the commonness in their biological basis of in cancer treatment. Despite the great efforts in the research field, the understanding of the antitumor mechanism of health-strengthening medicine is not clear enough [20,21]. The solution is constrained by the following three interrelated factors: the complex composition of TCM, the lack of target information of TCM, and the complex biological system involved in cancer development. To further promote the application of TCM in the treatment of cancer, proposing a comprehensive analysis strategy for exploring the impact of TCM from a holistic point of view is urgently needed.

An increasing amount of evidence indicates that TCM may exert therapeutic effects by targeting a variety of biomolecules [22]. However, due to the complexity of the ingredients and the limitations in the application of experimental methods, the targets of many TCM compounds are still unclear [23]. It has been proposed that TCM formulae and herbs impact the network of targets in complex diseases, such as cancer [24,25,26,27], and researchers may investigate the systemic effects of drugs on biological networks. The systematic concept is consistent with the multitarget characteristic of TCM and makes it suitable for studying the complex mechanism of TCM [28,29]. The advent of the big data era, the continuous accumulation of omics data, and the progress of bioinformatics methods provide strong support for the development of network pharmacology [30]. As a core concept in network pharmacology, network targets have changed the current research mode of “single target” and provided a potential research strategy for analyzing the biological basis of TCM from the perspective of networks and guiding the discovery of new active ingredients in TCM [31].

The development of high-throughput transcriptional assay technologies provides researchers with a comprehensive viewpoint for exploring the effect of compounds on gene expression. High-throughput methods are an integral part of pharmacological studies and have led to many achievements in biomedical fields [32]. High-throughput experimental methods, together with other genomic technologies, enables a comprehensive and systematic approach to the biological basis of medicine. TCM is widely recognized as a holistic treatment to diseases [13] and the mechanisms of TCM in cancer treatment are still unclear. Hopefully, the development of high-throughput methods will shed light on deciphering the comprehensive mechanism of TCM in cancer treatment. Here, a unique high-throughput screening strategy by high-throughput sequencing, referred to as HTS^2^ [33], was adopted for investigating the biological basis of 166 TCM compounds in cancer simultaneously. In the HTS^2^ assay, we added the compound library to the cell line and obtained a large-scale and quantitative transcriptional profiling in cells by detecting the signals of the designed gene probes.

To approach the systematic mechanisms of TCM compounds for cancer treatment, we combined network pharmacology prediction methods with HTS^2^ assay methods in our data analysis process. In this research, the Kyoto Encyclopedia of Genes and Genomes (KEGG) pathways involved in the antitumor or immunological activities of the TCM compounds are identified [34]. Taking advantage of the prediction and assay results, we conducted a systematic investigation on the antitumor mechanism of compounds in the two types of herbs (health-strengthening and pathogen-eliminating herbs), compounds in the same herb, and compounds that regulate the same pathway. Our study also revealed a potential bioactive compound, galloylpaeoniflorin, for cancer therapy, which may exhibit its efficacy via both regulating immune-related and antitumor pathways.

Together, by combining target prediction and high-throughput assay, this study proposed a systematic overview on the biological basis underlying the pharmacological effects of health strengthening herbs in cancer treatment. Despite the need for further investigation, it was indicated that health-strengthening herbs may provide researchers with a valuable candidate library for tumor immune regulatory and tumor preventive drug development.

## 2. Results

### 2.1. The Prediction and Examination of Potential Targets of by Literature Mining and the HTS^2^ Assay

Due to the complex composition of TCM and the lack of corresponding target records, the potential target lists of TCM compounds were obtained by using a computational prediction method, drugCIPHER-CS [12]. Literature mining based on text searching was conducted to verify the reliability of the target prediction results. The co-occurrence of the compound and target appearing in one or more abstracts was used to define the association between them. For each investigated TCM compound, we searched the PubMed database by its name in the abstracts and counted the total item number of the search results. Since the numbers of the related reports of the different TCM compounds varied greatly, which would influence the following analysis results (e.g., the percentage of predicted targets verified by literature), we only selected the compounds with adequate related reports (total item number ranging from 500 to 1000) for the verification of target prediction results. All the related abstracts of the selected compounds were downloaded, and text-processing codes were programed for extracting the biomolecules mentioned in the abstracts. The results from literature mining were then verified via manual examination by deleting the false positive responses.

**Figure 1:**
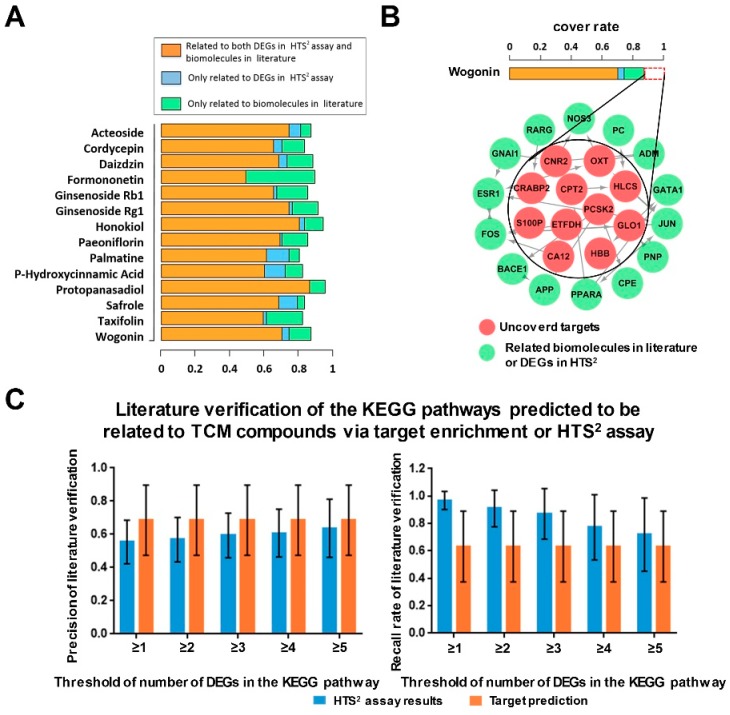
Evaluation of the target prediction results based on the literature and the HTS^2^ assay results. (**A**) The ratio of the predicted targets of the TCM compounds covered by the literature and the assay results. The cover rate was calculated as $\frac{\left|\mathrm{The}\;\mathrm{predicted}\;\mathrm{targets}\;\mathrm{related}\;\mathrm{to}\;\mathrm{the}\;\mathrm{reported}\;\mathrm{targets}\;(\mathrm{or}\;\mathrm{DEGs})\right|}{\left|\mathrm{The}\;\mathrm{predicted}\;\mathrm{targets}\right|}\times 100\%$. (**B**) Some targets of wogonin, which were not related to biomolecules in the literature or the DEGs in the HTS^2^ assay, were in the same pathway or connected via the protein–protein interaction (PPI) in STRING with the biomolecules in the literature or the DEGs in the HTS^2^ assay for wogonin. (**C**) Literature verification of the KEGG pathways related to the TCM compounds for cancer treatment with target enrichment and HTS^2^ evidence. Error bars represent the precision and recall rates of different TCM compounds. The precision was $\frac{\left|\mathrm{The}\;\mathrm{predicted}\;\mathrm{relevant}\;\mathrm{pathways}\;\mathrm{related}\;\mathrm{to}\;\mathrm{the}\;\mathrm{reported}\;\mathrm{relavant}\;\mathrm{pathways}\right|}{\left|\mathrm{The}\;\mathrm{predicted}\;\mathrm{relevant}\;\mathrm{pathways}\right|}\times 100\%$. The recall rate was $\frac{\left|\mathrm{The}\;\mathrm{predicted}\;\mathrm{relevant}\;\mathrm{pathways}\;\mathrm{related}\;\mathrm{to}\;\mathrm{the}\;\mathrm{reported}\;\mathrm{relavant}\;\mathrm{pathways}\right|}{\left|\mathrm{The}\;\mathrm{reported}\;\mathrm{relevant}\;\mathrm{pathways}\right|}\times 100\%$. Data represent mean ± SD.

After literature mining, we obtained the biomolecules mentioned in the abstracts related to the compounds in Figure 1A. Additionally, the differentially expressed genes (DEGs) after treatment with these TCM compounds in the cell line were achieved using the HTS^2^ assay. We examined whether a predicted target was directly or indirectly related with the biomolecules in the literature or DEGs in the HTS^2^ assay. The indirect relationship was established if the predicted target was in the upstream in a KEGG pathway of the biomolecules in the abstracts or DEGs, or if they were related by protein–protein interaction (PPI) in the HPRD, BIND, IntAct, MINT, or OPHID database [35,36,37,38,39]. The cover rate demonstrated in Figure 1A stands for the percentage of the predicted targets supported by literature or the HTS^2^ assay. The cover rate was calculated as $\frac{\left|\mathrm{The}\;\mathrm{predicted}\;\mathrm{targets}\;\mathrm{related}\;\mathrm{to}\;\mathrm{the}\;\mathrm{reported}\;\mathrm{targets}\;(\mathrm{or}\;\mathrm{DEGs})\right|}{\left|\mathrm{The}\;\mathrm{predicted}\;\mathrm{targets}\right|}\times 100\%$. The results (Figure 1A) indicated that the predicted target lists of TCM compounds for cancer basically covered 75%–90% of the biomolecules in the literature and had a relatively strong reliability. In addition, some potential targets were verified by the HTS^2^ experimental results, which demonstrated that the HTS^2^ assay may be an alternative method for exploring the novel biological functions of TCM compounds.

In Figure 1A, we found that 87% of the predicted targets of wogonin, a representative compound from *Radix Scutellariae* (*Huang Qin*), were supported with literature or assay evidence. As for the other 13% of the potential targets of wogonin predicted by drugCIPHER-CS, some of them were connected to the biomolecules in the literature or the DEGs in the HTS^2^ assay by an additional indirect mapping, as shown in Figure 1B. The indirect mapping in Figure 1B represented protein–protein interaction (PPI) in the STRING database [40] or relations via pathways in KEGG.

Next, to further analyze the mechanism of the action of the compounds, we performed KEGG pathway enrichment analysis based on the target prediction results. We checked the enrichment *p*-values of the targets in 26 cancer hallmarks (immune-excluded) and immune-related KEGG pathways. The literature verification of the pathways with HTS^2^ or enrichment evidence was conducted manually by reading the papers in the PubMed database. If a significantly enriched pathway was related to the bioactivity records in the published papers in the PubMed database [41] or included enough DEGs (the cut-off value was set as between one to five DEGs and the robustness of the threshold was measured in Figure 1C) in the HTS^2^ assay results, then it would be considered as a predicted related pathway with supports from literature records or HTS^2^ assay results. As shown in Figure 1C, a series of cut-off values (from one to five DEGs in a pathway in the HTS^2^ assay) was set to examine the robustness of the literature verification results. Our results indicated that the cut-off values had little influence on the precision rate for the related pathways determined using an HTS^2^ assay, and the recall rate scaled from 97% to 72% as the cut-off changed. The precision and recall rate of the significantly enriched KEGG pathways (*p* < 0.05, false discovery rate (fdr) adjusted) were approximately 60% and 70%, respectively, which indicated the reliability of the pathway prediction results. By comprehensively considering both the precision and recall results, we selected the pathways with three or more DEGs in the HTS^2^ assay as the ones with support from the assay data for further investigation.

### 2.2. Target Prediction and Assay Results Indicate that the Two Types of TCM Herbs May Regulate Several Key Biological Processes in Cancer Treatment, Including Antitumor and Immune Modulation

**Figure 2:**
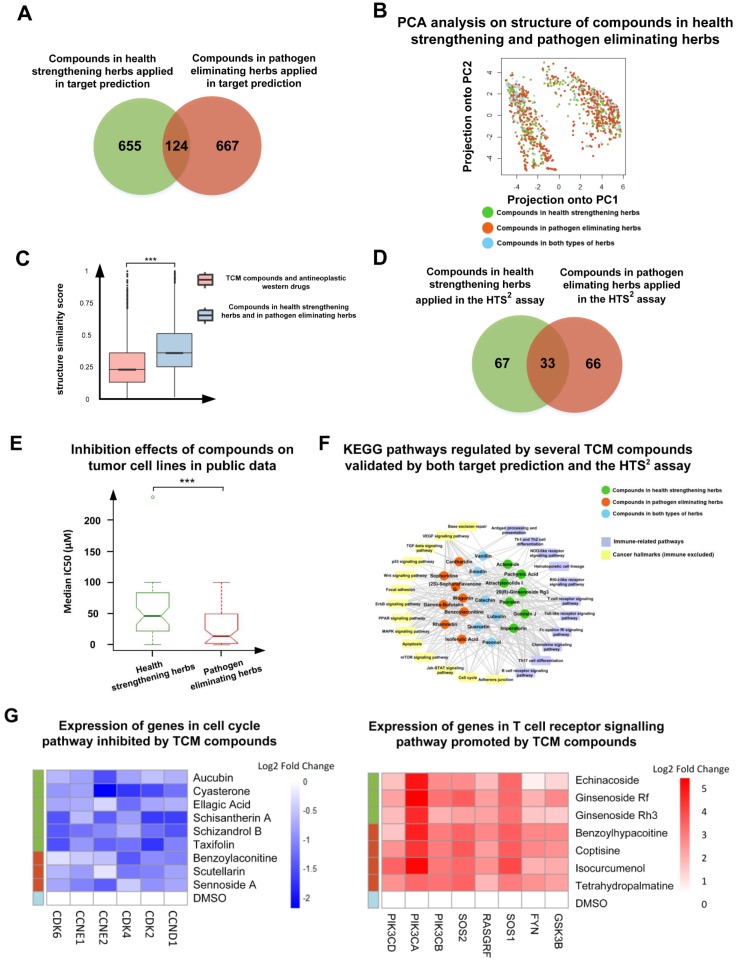
An overview of types of TCM applied in the research on their structures, inhibitory effects on tumor cells, and regulated bioactivities, based on public data, target prediction, and the HTS^2^ assay. (**A**) The number of compounds in the two types of TCM herbs in cancer treatment applied in the target prediction. (**B**) PCA analysis on structure of compounds in the two types of herbs in cancer treatment. (**C**) The structure similarity comparison between the compounds from health-strengthening herbs and pathogen-eliminating herbs and that between the TCM compounds and antineoplastic Western drugs via Tanimoto coefficients. Data represent median ± interquartile range. Statistical analysis was performed using a Kolmogorov–Smirnov (KS) test. *** *p* < 0.001. (**D**) The number of compounds from the two types of TCM herbs applied in the HTS^2^. (**E**) The inhibitory effects of compounds in the two types of herbs on proliferation of tumor cell lines in public bioactivity databases. Data represent median ± interquartile range. Statistical analysis was performed using a Wilcoxon rank sum test. *** *p* < 0.001. (**F**) The regulated immune and cancer hallmarks (immune-excluded) pathways of several TCM compounds supported using target prediction and the HTS^2^ assay. (**G**) Expression data of several genes in the cell cycle and T cell receptor signaling pathways after TCM compound treatments.

Historically, TCM encompasses a two-way philosophy in cancer treatment in that it is involved in both health strengthening and pathogen elimination. TCMs applied in curing cancer are classified as health-strengthening or pathogen-eliminating herbs according to their therapeutic effects. However, the biological functions of the two types of anti-cancer TCMs have not yet been elucidated. Therefore, we hoped to identify the regulated pathways of both types of TCM herbs by taking advantage of network pharmacology prediction and the HTS^2^ assay. As shown in Figure 2A, 1446 compounds were selected for target prediction, including 655 compounds in health-strengthening herbs, 667 compounds in pathogen-eliminating herbs, and 124 compounds in both types of herbs. In Figure 2B, the structural similarities among the compounds were measured by applying a principle component analysis (PCA) method to the compound 2-D structure information from the ChEMBL database [42]. A total of 881-dimensional CADD Group Chemoinformatics Tools and User Services (CACTVS) substructures in PubChem [43] were adopted to encode the structures of the investigated TCM compounds into binary vectors. The PCA analysis was conducted by using the princomp function in the R packages stats v3.2.2 (RStudio, Boston, MA, USA) under the environment of RStudio v1.1.447 [44]. As demonstrated in Figure 2B, the compounds in health-strengthening and pathogen-eliminating herbs may contain similar substructures. This result was consistent with the fact that health-strengthening and pathogen-eliminating herbs contained multiple compounds with common herbal chemical types, such as saponins, flavonoids, and alkaloids. For further unveiling the structural basis of the two clouds of compounds in Figure 2B, we examined the 10 CACTVS substructures that contributed the most to the first and the second feature vectors in the PCA analysis. The selection of the feature vectors was consistent with the two dimensions depicted in Figure 2B. The contributions were determined by the absolute values of the coefficients of the first two feature vectors. C:CC=C, C=C-C:C, O-C-C:C, C:C-C:C, and O-C-C:C-C were the five strongest positive features, and ≥ 3 any ring size 6, ≥ 4 any ring size 6, C(-C)(-C)(-H)(-O), C(-C)(-H)(-O), and [#1]-C-O-[#1] were the five strongest negative features. Therefore, the left cloud of compounds was more likely to contain the substructures among the negative features and the right cloud of compounds was more likely to contain the substructures among the positive features in Figure 2B. As shown in Figure 2C, it was found that the average structure similarity score of the specific compounds in the two types of herbs was significantly higher than that between the TCM compounds and antineoplastic Western drugs (*p* < 0.001). The structure similarity analysis was conducted by calculating Tanimoto coefficients [45] between the 881-dimensional CACTVS substructures of the compounds.

Among the 1446 TCM compounds applied in target prediction, 166 compounds were used in the HTS^2^ assay, including 67 compounds in health-strengthening herbs, 66 compounds in pathogen-eliminating herbs, and 33 compounds in both types of herbs (Figure 2D). In the HTS^2^ assay, approximately 3000 HCT116 colorectal cancer cells were seeded in each well of a 384-well plate for 24 h. Then the TCM compound library were added to the cells for 24 h to achieve a transcriptional profile after compounds treatment. Eight dimethyl sulfoxide (DMSO) replicates were also added in the wells as controls. After obtaining the gene probe reads, we performed gene expression normalization by using 18 stable genes in in colorectal cancer (GSE44076, GSE44861, GSE53295, and GSE53965 in the Gene Expression Omnibus (GEO) database [46]). The normalized expression of a gene was defined as the reads of the gene probe divided by the median number of the reads of 18 housekeeping genes. The fold change was calculated as the normalized gene expression after the drug treatment divided by the median number of normalized gene expression after the eight DMSO replicates treatment. For each TCM compound, genes with a fold change > 2 were considered DEGs.

To explore the inhibitory effects on proliferation in the different cell lines of compounds from health-strengthening and pathogen-eliminating herbs, we analyzed the half-maximal inhibitory concentration (IC50) and the half-maximal inhibitory concentration on cell growth (GI50) data of the TCM compounds collected from the ChEMBL database or literature. The median IC50 (or GI50) was calculated as the median number of all the human cell line specific experiments in ChEMBL and literature records after the TCM compound treatment. These two metrics were considered as the same and were merged in the analysis. Even though the compounds from pathogen-eliminating herbs exhibited the lower median IC50s (or GI50s) than compounds from health-strengthening herbs in Figure 2E, the results indicated that compounds in health-strengthening herbs might be anti-proliferative to tumor cells. The IC50 and GI50 data were also adopted as reference concentrations in the (3-(4,5-dimethylthiazol-2-yl)-5-(3-carboxymethoxyphenyl)-2-(4-sulfophenyl)-2H-tetrazolium (MTS) assay, a cell survival rate measurement assay, and these bioactivity data were then applied in setting the concentrations of the TCM compounds in the HTS^2^ assay after the manual adjustment.

In Figure 2F, target prediction and HTS^2^ assay suggested that some compounds may regulate immune and cancer hallmarks (immune-excluded) pathways simultaneously. As shown in Figure 2G, in the cell cycle pathway, the expression of the CDK4, CDK6, and CCND1 genes in the HTS^2^ assay were reduced after treatment with several compounds in the two types of herbs, while the gene expressions in T cell receptor signaling pathways were upregulated after treatment with several TCM compounds from the two types of herbs. The percentage of the experimental compounds in health-strengthening herbs with predicted targets enriched in the immune-related pathways was significantly higher than that in pathogen eliminating herbs (e.g., 37.3% and 22.7%, respectively, in antigen processing and presentation, and 65.7% and 47.0%, respectively, in natural killer cell mediated cytotoxicity) (Fisher exact test, *p* < 0.05). In Table 1, the regulated pathways of several compounds from health-strengthening and pathogen-eliminating herbs predicted using target enrichment and the HTS^2^ assay results were listed. These results indicated that TCM herbs, no matter their therapeutic classification, may exhibit therapeutic effects in cancer treatment via both immune regulation and other antitumor functions.

### 2.3. HTS^2^ Assay Results Show that Health-Strengthening Medicine May Regulate Tumor Immunity Via Promoting NK Cell Activity and Tumor Cell Antigen Presentation

**Figure 3:**
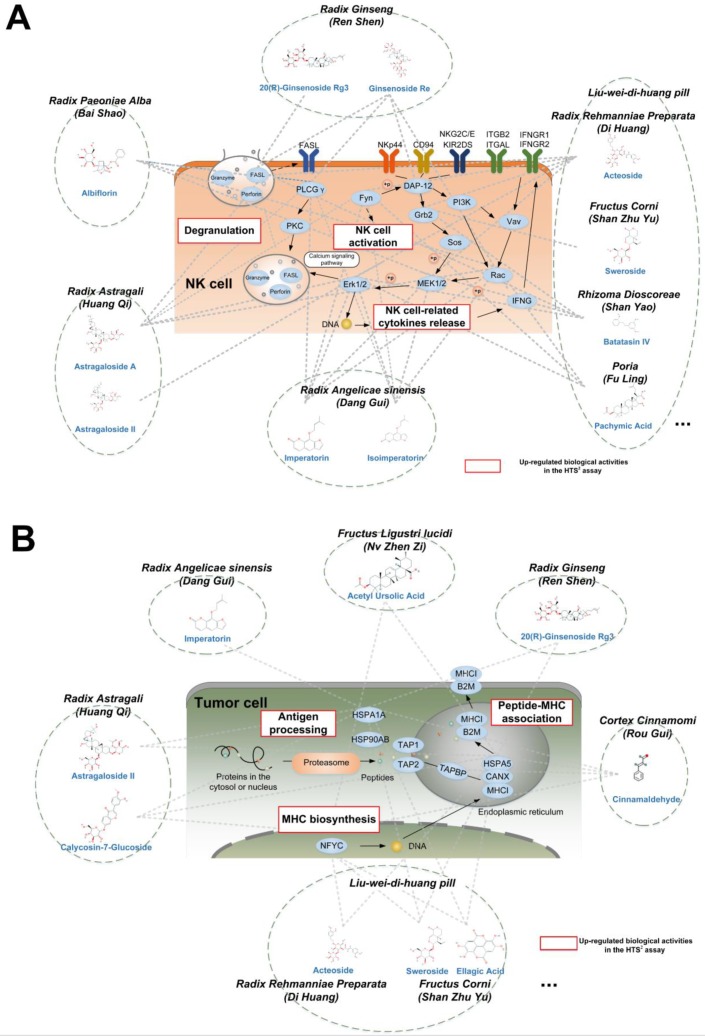
The regulatory effects on immune-related pathways induced by a group of compounds from health-strengthening herbs. The dotted lines linked the TCM compounds and the DEGs after compound treatment in the HTS^2^ assay. (**A**) The HTS^2^ assay results showed that compounds from health strengthening herbs upregulated the biomolecules in the NK cell mediated cytotoxic pathway. (**B**) The HTS^2^ assay results showed that compounds from health strengthening herbs upregulated the biomolecules involved in the MHC-I antigen processing and presentation pathway.

NK cells recognize and kill tumor cells containing the mutated gene fragments [47]. In the tumor environment, the NK cell activity is inhibited by the biological function of the tumor cells [48]. In Figure 3A, some TCM compounds from health-strengthening herbs with the potential biological functions of promoting NK cell activity were listed at the top, as indicated by the HTS^2^ assay results. For instance, ginsenoside Re, a compound in *Radix Ginseng (Ren Shen)*, was suggested to exert an NK cell activation effect by upregulating the expression of genes involved in degranulation and NK cell-related cytokines release.

Antigen processing and presentation plays an important role in tumor immunity [49]. In the tumor environment, the antigen presentation functions of tumor cells are relatively suppressed [50]. Therefore, the immune system may not fully recognize and kill tumor cells. The analysis of the HTS^2^ assay results revealed that several health strengthening herbs may comprehensively increase the level of antigen presentation in tumor cells by promoting intracellular synthesis of main histocompatibility complex class I (MHC-I) molecules, improving antigen processing efficiency, and combining tumor cell biopeptides with MHC-I molecules (Figure 3B). For example, sinnamaldehyde, a compound in *Cortex Cinnamomi (Rou Gui)*, was indicated to upregulate antigen processing and MHC biosynthesis related genes, as shown in Figure 3B. Additionally, as demonstrated in Figure 3B, some compounds that may enhance the NK cell activities may also improve the levels of antigen processing and presentation in tumor cells.

### 2.4. Target Prediction and HTS^2^ Assay Results Show that Compounds in the Same Herb May Exhibit Different Patterns in Modulating Antitumor or Immune Processes

**Figure 4:**
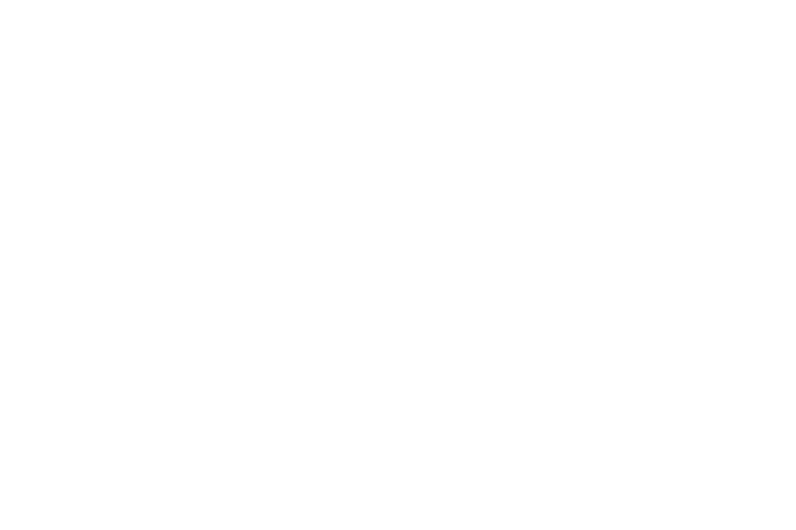
The HTS^2^ assay results indicated that within the same pathway, compounds from the same herb may influence gene expression in the same cancer hallmarks (immune-excluded) or immune-related KEGG pathway in the same or opposite directions, as shown in the three boxes. (**A**) Compounds from the same herb may simultaneously upregulate the gene expression in the same pathway. (**B**) Compounds in the same herb may simultaneously downregulate the gene expression in the same pathway. (**C**) Compounds from the same herb may regulate the gene expression in the same pathway in opposite directions.

To further identify how the combinations of the TCM compounds in the same herb may regulate the same biological process, we extracted the HTS^2^ results of the TCM compounds that were predicted to regulate the same pathway. Next, we examined the expression data of the genes in the predicted regulated pathways. After the integration of the HTS^2^ results from the TCM compounds and the gene information in the KEGG pathways, we concluded that the TCM compounds in the same herb may have interactions in several patterns in regulating the same KEGG pathway. As shown in Figure 4, the selected TCM compounds may regulate the same pathway in the same or opposite directions to induce the final effects, as indicated by the HTS^2^ assay results. For instance, kaempferol and kaempferide are two compounds from the health-strengthening herb *Fructus Corni* (*Shan Zhu Yu*). After analyzing the HTS^2^ assay results of kaempferol and kaempferide, we discovered that they have similar molecular patterns for inhibiting cell cycles, as shown in Figure 4B. Additionally, paeonol and galloylpaeoniflorin, two compounds in *Radix Paeoniae Alba* (*Bai shao*), were indicated to influence gene expression in the mitogen-activated protein kinase (MAPK) signaling pathway in the opposite directions (Figure 4C). These results suggested that compounds in the same herb may exhibit similar or distinguished mechanisms in cancer treatment. The compounds with different mechanisms may have the potential bioactivities in treating different types of tumors.

### 2.5. Prediction and Assay Results Imply that Compounds in One Health-Strengthening Herb or a Single Compound May Exert Antitumor and Immune-Related Functions Simultaneously for Cancer Therapy

**Figure 5:**
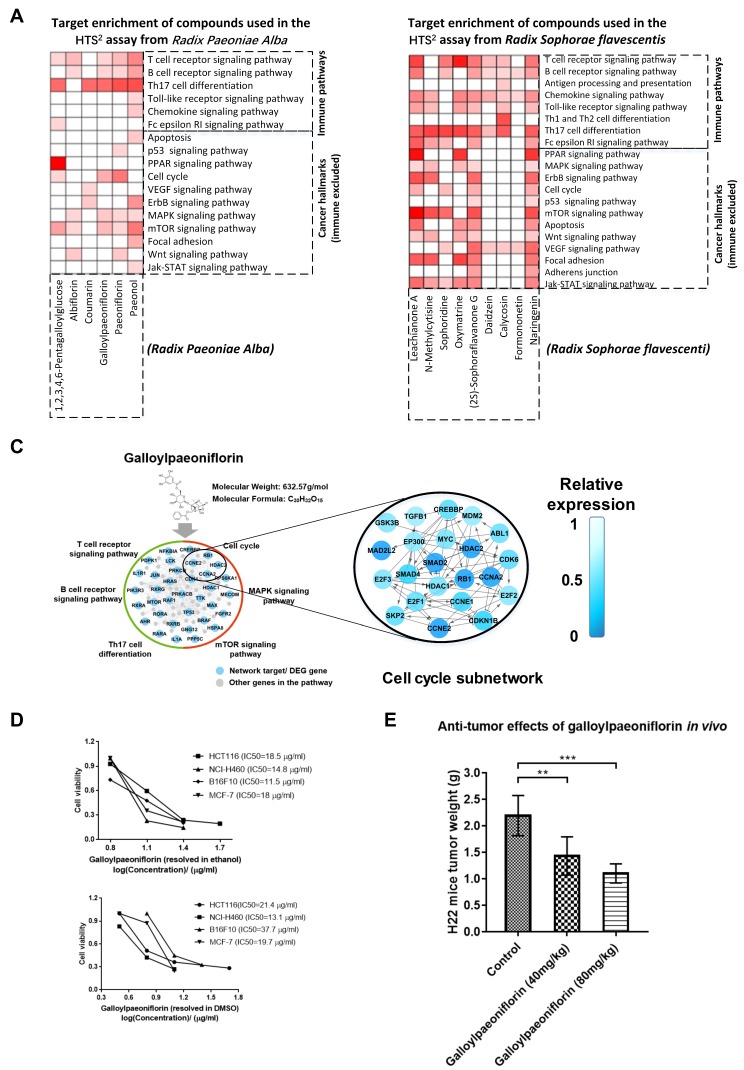
Identification of the immune regulatory and other antitumor biological functions of compounds from *Radix Paeoniae Alba* (*Bai Shao*) and *Radix Sophorae flavescentis* (*Ku Shen*). (**A**,**B**) The comprehensive functional characterization of the compounds in *Radix Paeoniae Alba* (A) or *Radix Sophorae flavescentis* (B) using the pathway enrichment analysis based on target prediction results. The white blanks represent pathways not regulated by the compounds according to HTS^2^ assay results. (**C**) The pathway regulation effects of galloylpaeoniflorin, a compound in *Radix Paeoniae Alba* (*Bai Shao*) using the target enrichment and HTS^2^ assay. A subnetwork representing the expression of the predicted targets of galloylpaeoniflorin in the cell cycle pathway was shown. (**D**) Inhibitory effects of galloylpaeoniflorin on tumor cell proliferation using the cell lines of several tumor types, as assessed using an MTT assay. The experiment was done once in triplicate. (**E**) Inhibitory effects of galloylpaeoniflorin on tumor growth in H22 mice. The assay was performed on seven BALB/c/nu nude female mice injected with the H22 tumor for each group. ** *p* < 0.01, *** *p* < 0.001, compared with the solvent control group. Statistical analysis was performed using multiple t tests. Data represent mean ± SD.

Considering the multicompound characteristics of TCM herbs, it is necessary to examine the biological functions of different compounds from one herb in treating cancer. As we know, *Radix Paeoniae Alba* (*Bai Shao*) and *Radix Sophorae flavescentis* (*Ku Shen*) are two typical health-strengthening and pathogen-eliminating herbs that are widely used for cancer treatment [2]. After target prediction and KEGG pathway enrichment, we extracted the mRNA expression induced by the compounds from *Radix Paeoniae Alba* and *Radix Sophorae flavescentis* in the HTS^2^ assay. The target enrichment results of the pathways with the assay evidence are shown in Figure 5A and Figure 5B. T cell receptor signaling pathway, B cell receptor signaling pathway, Th17 cell differentiation, MAPK signaling pathway, mTOR signaling pathway, and some other pathways were regulated by several compounds from *Radix Paeoniae Alba*, including 1,2,3,4,6-pentagalloylglucose, albiflorin, coumarin, galloylpaeoniflorin, paeoniflorin, and paeonol (Figure 5A). The target enrichment and HTS^2^ assay results suggested that all the compounds in Figure 5A might regulate both immune-related and other antitumor pathways. The similar pattern was found in the target enrichment results of the compounds from *Radix Sophorae flavescentis*. As shown in Figure 5B, nine compounds from *Radix Sophorae flavescentis* were selected for the HTS^2^ assay, and all of them may influence at least one immune-related pathway and one other antitumor pathway.

One of the compounds from *Radix Paeoniae Alba*, galloylpaeoniflorin, had no relevant antitumor records or any other biological activity records. The target prediction and HTS^2^ assay results indicated that galloylpaeoniflorin might regulate several immune-related pathways (i.e., T cell receptor signaling pathway, Th17 cell differentiation, and B cell receptor signaling pathway), and cancer hallmarks pathways (i.e., MAPK signaling pathway, cell cycle, and mTOR signaling pathway) (Figure 5C). For instance, the relative expression of genes in the cell cycle in the HTS^2^ assay was shown in the right part of Figure 5C, and the expression of the biomolecules listed in the subnetwork were reduced by galloylpaeoniflorin. Moreover, we examined the antitumor effects of galloylpaeoniflorin both in vitro and in vivo (Figure 5D,E). As shown in Figure 5D, galloylpaeoniflorin effectively inhibited the proliferation of several cell lines (IC50 < 40µg/mL), including HCT116 (a colorectal cancer cell line), B16F10 (a melanoma cell line), MCF-7 (a breast cancer cell line), and NCI-H460 (a lung cancer cell line) cells. Different solvents were applied (DMSO and ethanol) in the top and the bottom of Figure 5D and the results demonstrated robust inhibitory effects on the tumor cell lines induced by galloylpaeoniflorin. The in vitro inhibitory effects of galloylpaeoniflorin in Figure 5D on not only the HTC116 cells, which was adopted in the HTS^2^ assay, but also several cell lines of varied cancer types indicated its antineoplastic potentials in treating different tumors. The experiment in Figure 5D was done once in triplicate. Our in vivo results confirmed that the tumor weight of the H22 liver tumor mice was significantly reduced after the treatment with galloylpaeoniflorin (Figure 5E). Twenty-one BALB/c/nu nude female mice injected with H22 tumor were utilized for the in vivo assay.

## 3. Discussion

Health-strengthening medicine is seen as a representative application of the classical philosophy of TCM in cancer therapy. It is reported that, unlike Western medicine, which may exhibit direct killing effects on tumor cells, health-strengthening medicine is developed to treat cancer by systematically regulating the tumor microenvironment [51,52,53,54]. According to the traditional efficacy of TCM, health-strengthening herbs can be classified into different categories, such as yin-nourishing (*Zi-Yin*) herbs and qi-tonifying (*Yi-Qi*) herbs. In this research, as a first step, we treated health-strengthening herbs as a whole rather than the subcategories for the following network pharmacological analysis. Several biological processes may be involved in the regulatory effects of health-strengthening medicine, including some immune-related bioactivities. This finding is in accord with the previous studies on the comprehensive anti-tumor mechanisms of nuciferine, a compound from a yin-nourishing health-strengthening herb, *Nelumbo nucifera Gaertn* (*He Ye*) [55]. Additionally, some classical TCM formulae with the health-strengthening efficacy are clinically proven to enhance the innate immunological function (e.g., the killing abilities of NK cells) and the sensitivity of immune system to tumor cells [56,57]. These results were consistent with our findings that health-strengthening medicine might regulate the immune function in multiple aspects, including NK-cell-mediated cytotoxicity and antigen processing and presentation. However, the biological activities of health strengthening medicine have not been fully elucidated.

Here, based on the target prediction and the HTS^2^ assay results, we analyzed the potential bioactivities of compounds from health-strengthening herbs. Several pathogen-eliminating herbs were also included in the research paradigm for comparison. Our prediction and assay results suggested that compounds from both types of TCMs may regulate both immune and cancer hallmarks (immune-excluded) pathways. This finding was consistent with the multitarget characteristic of TCM compounds. We further investigated the differences between these two types of TCMs and discovered that the overall inhibitory effects of the compounds in pathogen-eliminating herbs on tumor cells were significantly stronger than those in health-strengthening herbs. The results were consistent with the traditional understanding that pathogen-eliminating herbs tend to target the tumor directly. However, the traditional therapeutic advantages of health-strengthening herbs on the immune system need to be further explored by adopting immunological experimental results, and our high-throughput assay was conducted on tumor cells. Additionally, we revealed a group of compounds from the same herb that influences pathway gene expression in the same or different directions. The compounds with opposite influences on pathway gene expression may be explained by the different underlying mechanisms in cancer treatment. Therefore, it was suggested that they may be applied for treating different types of tumors.

In addition, galloylpaeoniflorin, a compound from a health-strengthening herb *Radix Paeoniae Alba* (*Bai Shao*) was predicted to impact several pathways that are significant in tumor development, including T cell receptor signaling pathway, B cell receptor signaling pathway, cell cycle, and mTOR signaling pathway. The regulatory effects were further supported by the HTS^2^ gene expression profile of the related genes in the pathways after the treatment with galloylpaeoniflorin. In fact, the antitumor effects of *Radix Paeoniae Alba* and its total glucosides were found in recent research, and the mechanism may be related to the inhibitory effects on the cell cycle of tumor cells [58,59]. Notably, to the best of our knowledge, there is no activity record of galloylpaeoniflorin. Therefore, we examined and initially validated the antitumor effects of galloylpaeoniflorin both in vitro and in vivo. More work should be conducted for further evaluating the antitumor ability as well as unveiling the potential mechanism of galloylpaeoniflorin.

In this work, we divided cancer-related pathways into two categories: immune pathways and other cancer hallmarks for separate investigations. The distinction was made for the following reason: in the aspect of clinical treatment, immunotherapy and other approaches (e.g., targeted approaches and cytotoxic agents) are distinguished treatment options available in cancer treatment [60]. That was because the mechanisms that cancer immunotherapy are based on differ greatly from those of other approaches in cancer therapy [61]. Even though immune evasion is one of cancer’s hallmarks, it characterizes the responses using the immune system [62]. Also, this distinction was consistent with the Anatomical Therapeutic Chemical (ATC) drug classification, a drug classification system based on pharmacological and anatomical properties by WHO, in which antineoplastic (L01) and immunomodulating (L03 and L04) agents are independent drug catalogues [63].

To be mentioned, previous studies indicated that health-strengthening herbs may exert their therapeutic effects in cancer treatment in multiple aspects (e.g., immunity and metabolism) [14,15,16,17,18,19]. In this study, the immunological efficacy was selected as an example and more work is needed for a comprehensive understanding of their efficacy in other aspects, such as the metabolic regulation effects. Moreover, TCM syndrome (Zheng) is an essential concept in the TCM theory [64]. It is reported that TCM syndromes correlates with treatment response to TCM in cancer therapy [65]. Therefore, future studies on the therapeutic effects of health strengthening herbs cancers with different TCM syndromes would further promote the understanding of the biological basis of health-strengthening herbs.

According to the analysis results in this study, health-strengthening herbs may exhibit both immune-regulatory and antitumor effects. Taking into consideration the generally weaker antitumor effects (IC50 or GI50) in vitro of compounds in health strengthening herbs than that in pathogen-eliminating herbs in public records, the high percentage of selected compounds in health-strengthening herbs related to immune-related pathways, and the good safety of health-strengthening herbs, it was indicated that health-strengthening herbs may have more pharmacological potential in preventing tumors and improving a tumor-immune microenvironment, compared to directly killing tumor cells.

In contrast to previous studies, our study features the usage of high-throughput computational and experimental methods for a more comprehensive understanding of underlying mechanism of health-strengthening herbs. HTS^2^ may significantly promote the parallel processing of candidate compounds and genes, and has been applied in drug screening [66]. However, this study only provided in vitro large-scale experimental results of the HTS^2^ assay on one cell line, HCT116, and it does not represent cells of the immune system. Therefore, most of the results in this manuscript are hypothesis-generating and more experimental studies are needed to further explore the bioactivities regulated by TCM compounds. Still, the research strategy in this study does provided avenues for large-scale experimentation. Hopefully, our study may help reveal the biological basis of health-strengthening herbs, a characteristic herb type which has been used for a long period by TCM practitioners, and shed light on the future researches on anti-tumor drugs by unveiling the wisdom of TCM.

## 4. Materials and Methods

### 4.1. TCM Compounds Data Preparation

We collected compound information of 47 TCM herbs (including 22 health-strengthening herbs and 25 pathogen-eliminating herbs for comparison) that are widely used in cancer therapy from the Chinese national medical insurance catalogue and commonly used prescriptions (e.g., Liu-wei-di-huang pill, *Buzhong Yiqi* decoction, and *Sijunzi* decoction) (Supplementary Table S1). The 22 health-strengthening herbs include different categories, such as yin-nourishing (*Zi-Yin*) herbs and qi-tonifying (*Yi-Qi*) herbs, and the 25 pathogen-eliminating herbs include categories, such as heat clearing and detoxifying (*Qing-Re-Jie-Du*) herbs, and blood activating and stasis dissolving (*Huo-Xue-Hua-Yu*) herbs, according to the traditional classification based on TCM efficacy. Together, 1446 TCM compounds with PubChem records were collected, including 655 compounds from health-strengthening herbs, 667 compounds from pathogen-eliminating herbs, and 124 compounds from both types of herbs.

### 4.2. Analysis Workflow Based on Network Pharmacology

**Figure 6:**
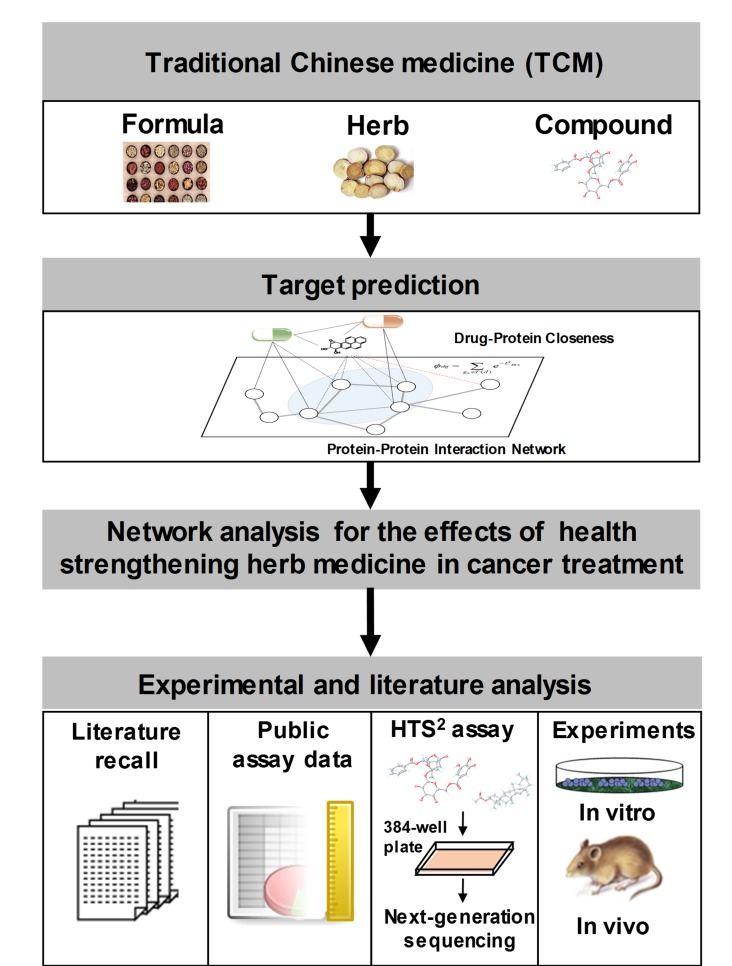
The network analysis workflow for understanding the effects of health-strengthening medicine compounds in cancer treatment.

In this study, we proposed an approach based on network pharmacology to study the antitumor mechanisms of health-strengthening medicine. The network pharmacology approach was applied for predicting the potential targets of the TCM compounds and to visualize the analysis results as networks in this manuscript. After the TCM compounds data preparation, we predicted the potential targets of the TCM compounds by utilizing an algorithm based on the correlation between the pharmacological network and genomic network. The prediction results were analyzed via literature mining, the HTS^2^ assay, public assay data, and in vitro and in vivo experiments. Taking advantage of the prediction and analysis results, we analyzed the molecular functional patterns of health-strengthening herbs (Figure 6).

### 4.3. Literature Mining

The literature mining was performed via text searching and no algorithm was applied in the process. By literature mining, we aimed to obtain the related biomolecules for each TCM compound and to compare the results with the predicted targets. The co-occurrence of compound and target appearing in one or more abstracts was used to define the association of them.

We searched the PubMed database using the name of each TCM compound in the abstracts and recorded the number of returned search results. In this study, we selected the compounds with an adequate number of search results (between 500 to 1000) for further analysis. The interval was necessary because the number of the related literature varied greatly, and this would have impacts on the following analysis (e.g., the percentage of predicted targets verified by literature records). All the related abstracts of the selected compounds were downloaded, and text-processing codes were programed for extracting the biomolecules mentioned in the abstracts. If a biomolecule co-occurred in the abstract with the compound name, then we considered that the biomolecule was related to the compound.

The results from the literature mining were then verified via a manual examination by deleting the false positive responses. Then, the results were used as the verification set for target prediction and HTS^2^ assay results.

### 4.4. Target Prediction for the TCM Compounds Applied in Cancer Treatment

The potential targets of the TCM compounds were predicted by drugCIPHER-CS [24] using Matlab 2016a (MathWorks, Natick, MA, USA) [67], a network-based target prediction method. Using a liner regression model, this method correlates pharmacological and genomic spaces for predicting the drug targets. In this method, the likelihood of a candidate compound targeting a specific protein can be described as a concordance score between the structural similarity vector of the candidate compound and drugs in DrugBank [68] and the drug-protein closeness vector based on PPI. According the article on drugCIPHER-CS, the accuracy of target prediction is 77.3% when the top 100 biomolecules were chosen to form a potential target profile, as measured using cross-validation. Therefore, in this study, the top 100 biomolecules in the prediction result list were selected as the potential target list of each TCM compound. The PPI network was constructed by combining the PPI information recorded in HPRD, BIND, IntAct, MINT, and OPHID in May 2011 [35,36,37,38,39], and it contained 137,037 PPIs for 13,388 human proteins. Drug–protein interactions were retrieved from DrugBank associated to the PubChem database in May 2015 [43]. Drug structural similarity vectors were the Tanimoto coefficients [45] based on 881-dimensional CACTVS substructures in PubChem.

Moreover, to measure the reliability of target prediction results, biomolecules mentioned in the literature and DEGs in HTS^2^ results were collected. If a predicted target is directly or indirectly related to a biomolecule which co-occurred with the compound in the literature or to a DEG in the HTS^2^ assay after treatment with the compound, it is considered to be supported by the literature mining and HTS^2^ assay. The indirect relationship was established if the predicted target in in the upstream in a KEGG pathway of the biomolecules in the abstracts or DEGs, or if they are related via PPI. The cover rate was calculated as $\frac{\left|\mathrm{The}\;\mathrm{predicted}\;\mathrm{targets}\;\mathrm{related}\;\mathrm{to}\;\mathrm{the}\;\mathrm{reported}\;\mathrm{targets}\;(\mathrm{or}\;\mathrm{DEGs})\right|}{\left|\mathrm{The}\;\mathrm{predicted}\;\mathrm{targets}\right|}\times 100\%$.

### 4.5. KEGG Pathway Enrichment Analysis

We performed the KEGG pathway enrichment analysis for the predicted targets of the TCM compounds applied in cancer therapy in order to identify their biological functions. We used a hypergeometric test for enrichment analysis. We performed target enrichment under the background of 13388 human proteins and checked the *p*-values of the pathways to see if they were significantly enriched. The enrichment *p*-values of 26 pathways from cancer hallmarks and immune-related pathways in the KEGG database were examined. The enrichment analysis was performed using RStudio v1.1.447 and an open source programming language, Ruby 2.3.0. The significantly enriched KEGG pathways (*p* < 0.05, fdr adjusted) were retained for further research.

### 4.6. Chemical Space Analysis

CACTVS substructures in PubChem were adopted in the chemical space analysis. In the analysis, we used 881-dimensional substructure binary vectors to encode the investigated TCM compounds. A PCA analysis was conducted using the princomp function in the R packages stats v3.2.2 under the environment of RStudio v1.1.447 [44]. The Tanimoto coefficients of the binary vectors of the TCM compounds in the two types of herbs and antineoplastic Western drugs were calculated.

### 4.7. Network Visualization

Network visualization was performed using Cytoscape v3.6.0 (National Resource for Network Biology, Bethesda, MD, USA) [69]. For visualization, the KEGG, HPRD, BIND, IntAct, MINT, and OPHID databases were used for providing pathway and PPI information.

### 4.8. Cell Culture

HCT116 (a colorectal cancer cell line), B16F10 (a melanoma cell line), MCF-7 (a breast cancer cell line), and NCI-H460 (a lung cancer cell line) cells were obtained from the cell center of Chinese Academy of Medical Sciences and Peking Union Medical College (CAMS and PUMC). The cells were cultured in an incubator with 5% CO_2_ at 37 °C. Dulbecco’s modified eagle medium (DMEM) containing 10% fetal bovine serum, 100 U/mL penicillin and 100 g/mL streptomycin were applied for the incubation.

### 4.9. The HTS^2^ Assay

The HTS^2^ assay is a high-throughput screening strategy that enables a large-scale and quantitative analysis of gene transcriptional profiles in cells [33]. In the HTS^2^ assay, approximately 3000 HCT116 colorectal cancer cells were seeded in each well of a 384-well plate for 24 h. After that, the TCM compounds library was added to the cells for another 24 h, including eight DMSO replicates as negative controls. The HTS^2^ assay was then conducted to obtain the transcriptional profiles of the designed gene probes. The cells were lysed in GentLys buffer (Nanopure, Beijing, China). The instrumentation of the HTS^2^ assay was an automated liquid handling system, which contained the Agilent Bravo automated liquid handling platform (Agilent, Santa Clara, CA, USA) and the Agilent bench robot (Agilent, Santa Clara, CA, USA). By RNA annealing, selection and ligation, the instrumentation automatically performed the HTS^2^ assay. Pooled pairs of oligonucleotides targeting selected gene probes by streptavidin-magnetic beads and the biotinylated oligo-dT were used. Then, the paired oligonucleotides were ligated using T4 DNA ligase and were amplified using Polymerase Chain Reaction (PCR). Using unique bar-coded primer in Illumina flowcells, the HTS^2^ assay allowed a high-throughput transcriptional profiling of up to 1400 genes from 2000 samples.

#### 4.9.1. Selection and Preparation of the TCM Compounds for HTS^2^

The selection of the TCM compounds for the HTS^2^ assay were performed considering their recorded biological activity data and the offer lists provided by the suppliers. The bioactivity data (IC50 and GI50) of the TCM compounds were collected from ChEMBL or via manual literature searching. The median IC50 and GI50 of a compound were achieved via calculating the median number in all human cell line specific experiments in ChEMBL and literature records. The two metrics, IC50 and GI50 were used as the same metrics in the analysis. Then, we chose the compounds with an adequate antitumor activity (median IC50 or GI50 < 100 µM) as candidate compounds for the HTS^2^ assay. The candidate compounds were further selected considering the product availability of the suppliers.

The TCM compounds were dissolved in DMSO. The concentrations were preliminarily set as the median IC50 and were adjusted afterwards based on the cell survival rate using MTS assay for the HCT116 cells to meet the standard for the HTS^2^ assay (cell survival rate > 70%). Detailed information about the compounds applied in the HTS^2^ assay (e.g., PubChem Compound Identifier (CID), supplier, and purity) was presented in Table S2.

#### 4.9.2. The Gene Selection and Probe Design of the HTS^2^ Assay

A total of 420 genes were selected to form a gene set for the HTS^2^ assay. The gene set contained immune-related and other antitumor-related genes. The selection of the 420 genes were achieved in three steps. First, the genes were selected from databases and by predictions to form a cancer-related gene lists, including genes in pathways in cancer (hsa05200) in KEGG, genes in colorectal cancer (hsa05210) in KEGG, the colorectal cancer related genes in OMIM (MIM Number: 114500) [70], the targets of antineoplastic drugs in DrugBank, and the colorectal cancer related genes predicted using CIPHER, a phenotype-gene network based algorithm [71,72]. The reason why we selected some genes related to colorectal cancer was that the HCT116 cell line applied in the following HTS^2^ assay was a colorectal cancer cell line.

Then, we referred to the public gene expression profiles in the GEO database for selecting a reliable set of genes with adequate expression levels. Here, we selected two profiles of samples from patients with colorectal cancer (GSE44076 and GSE44861) and two profiles of the HCT116 cell line (GSE53295 and GSE53965) for measuring the gene expression. A gene was selected if it had at least three of the above profiles in its expression data and if the expression ranked 10% to 60% in the detected gene set in at least one profile. Additionally, we selected 30 housekeeping genes that meet the following standards: (1) it was not in the cancer-related gene lists achieved in the first step; (2) the expression of the gene was detected in the four gene expression profiles (GSE44076, GSE44861, GSE53295, and GSE53965), and was not a DEG in any one of these profiles; and (3) the expression of the gene ranked 10% to 60% in the gene sets of at least one profile. The moderate ranking was to ensure that the gene expression was neither too high or too low, which may impair the credibility of the HTS^2^ assay results.

At last, the probes for the genes were designed, and 420 genes with efficient probes, were selected for the HTS^2^ assay, including 18 housekeeping genes. Sequences of 10 probes used in the HTS^2^ assay were provided in Table S3.

#### 4.9.3. HTS^2^ Data Processing

First, the reads were mapped to the probe sequences, and three mismatches for each were permitted. The raw experimental data of HTS^2^ assay after treatment with DMSO and several TCM compounds were provided in Table S4. The numbers in Table S4 were the reads of gene probes using the HTS^2^ assay, which represent the abundance of genes. The HTS^2^ data was normalized by the expression of 18 stable housekeeping genes. The normalized gene expression was computed with raw reads of the gene after the drug treatment and the median number of raw reads of 18 housekeeping genes after the drug treatment.

Second, to identify the DEGs for each TCM compound, we calculated the fold change of the tested genes as the normalized gene expression after the drug treatment divided by the median number of normalized gene expression after the eight DMSO replicates treatments. For each TCM compound, genes with fold change > 2 were considered DEGs.

Third, to evaluate the reliability of the transcriptional profile, we calculated the Pearson correlations among the normalized transcriptional data after treatment with the eight DMSO replicates. The results are demonstrated in Figure S1. The correlations ranged from 0.84 to 0.99, which indicated the reliability of the assay results.

### 4.10. Cell Viability Assay

For exploring the antitumor effects of galloylpaeoniflorin, we further performed an MTT cell viability assay on different tumor cells. The HCT116, B16F10, MCF-7, and NCI-H460 cell lines were seeded in a 96-well plate before the drug treatment. Various concentrations of galloylpaeoniflorin dissolved in DMSO and ethanol respectively were added to the cells after incubation for 24 h. To assess the IC50s of the cell lines, the MTT cell viability assay was conducted after incubation for another 120 h. The IC50s were achieved by fitting the dose-response curve. The MTT assay was done once in triplicate.

### 4.11. Animal Studies

Twenty-one six-weeks-old BALB/c/nu nude female mice obtained from the Vital River Laboratories (Vital River Laboratories, Beijing, China) and were used for the xenograft experiments. H22 cells were injected into the left flank of the mice. When the tumor volume reached the size of 100–250 mm^3^, the mice were randomly separated into three groups and were administered an oral dose of galloylpaeoniflorin (40 mg/kg/day or 80 mg/kg/day) or vehicle control (1 × solution with cremophor EL/ethanol/water (12.5:12.5:75)). The mice were sacrificed at the end of the treatment period. The tumor volume was measured and weighted for the analysis.

The animal experiments were conducted in accordance with the guidelines for the care and use of laboratory animals. The work was approved by the Animal Care Committee of Chinese Academy of Medical Sciences and Peking Union Medical Colleges (Beijing, China) (Permit Number: SYXK 2015-0025).

### 4.12. Statistical Analysis

Data are shown as the means ± standard deviation (SD) (Figure 1C and Figure 5E) and median ± interquartile range (Figure 2C and Figure 2E). Multiple types of data were used in the manuscript and various statistical analysis methods were applied. Statistical analysis was performed using Kolmogorov-Smirnov (KS) test in Figure 2C, Wilcoxon rank sum test in Figure 2E and Student *t*-tests in Figure 5E. The significance levels were set at * *p* < 0.05, ** *p* < 0.01, and *** *p* < 0.001.

## 5. Conclusions

In conclusion, in this study, we performed a network-based analysis of health-strengthening medicine by integrating a series of methods, including target prediction, literature mining, the HTS^2^ experiment, and some low-throughput assays. The expression levels of 420 genes, associated with tumor growth and immune functions, were detected after a parallel treatment of 166 TCM compounds. By combining evidence from different sources, we helped further uncover the biological basis of health-strengthening medicine. We concluded that health-strengthening herbs, might regulate both immune-related and antitumor pathways, similar to pathogen-eliminating herbs. A typical case was demonstrated by *Radix Paeoniae Alba (Bai Shao)*, a health-strengthening herb widely used in TCM cancer treatment. Galloylpaeoniflorin, a compound from *Radix Paeoniae Alba*, was predicted to regulate several essential biological processes in cancer development, and its antitumor effect was preliminarily proven both in vivo and in vitro. Additionally, some TCM compounds in the same herb were indicated to regulate pathway gene expression with similar or different patterns, suggesting the urgent need for further in-depth studies on TCM prescriptions. For instance, acetyl ursolic acid and specnuezhenide, two compounds in a health strengthening herb *Fructus Ligustri lucidi (Nv Zhen Zi)*, both upregulated gene expressions in T cell signaling pathway in HTS^2^ assay. In summary, this study provided a new research strategy for explaining the biological basis of health-strengthening herbs, and further suggested the tumor immune regulatory and tumor preventive potentials of health-strengthening herbs.

## Figures and Tables

**Table 1:** Several KEGG pathways predicted to be regulated by compounds from health-strengthening and pathogen-eliminating herbs using target enrichment and the HTS^2^ assay.

KEGG Pathway	Herb Type	Herb	Compounds	Enrichment *p*-value	DEG Number
Apoptosis	*Fu-Zheng*	*Radix Angelicae sinensis* (*Shan Yao*)	Batatasin IV Dioscin	2.1 × 10^−4^ 1.1 × 10^−2^	13 23
	*Qu-Xie*	*Rhizoma Curcumae* (*E Zhu*)	Curcumin Isocurcumenol	1.7 × 10^−3^ 1.7 × 10^−3^	11 31
vascular endothelial growth factor (VEGF) signaling pathway	*Fu-Zheng*	*Fructus Schisandrae* (*Wu Wei Zi*)	Schisanhenol Gomisin J	3.2 × 10^−3^ 3.2 × 10^−3^	14 7
	*Qu-Xie*	*Radix et Rhizoma Rhei* (*Da Huang*)	Chrysaron Rhein	2.3 × 10^−6^ 3.2 × 10^−3^	9 5
Cell cycle	*Fu-Zheng*	*Fructus Ligustri lucidi* (*Nv Zhen Zi*)	Ligustroflavone Specnuezhenide	2.4 × 10^−3^ 1.5 × 10^−3^	4 10
	*Qu-Xie*	*Cortex Moutan* (*Gan Chan Pi*)	Cinobufagin Telocinobufagin	1.5 × 10^−3^ 2.3 × 10^−4^	20 26
T cell receptor signaling pathway	*Fu-Zheng*	*Poria* (*Fu Ling*)	Pachymic Acid Poricoic Acid B	8.7 × 10^−7^ 7.8 × 10^−5^	5 8
	*Qu-Xie*	*Cortex Magnoliae officinali* (*Huang Qin*)	Baicalein Wogonin	7.8 × 10^−5^ 3.8 × 10^−3^	18 15
Toll-like receptor signaling pathway	*Fu-Zheng*	*Radix Astragali* (*Huang Qi*)	Astragaloside A Formononetin	4.1 × 10^−3^ 4.1 × 10^−3^	5 3
	*Qu Xie*	*Venenum Bufonis* (*Chan Su*)	Bufarenogin Cinobufagin	4.1 × 10^−3^ 4.1 × 10^−3^	15 20
Nucleotide-binding oligomerization domain (NOD)-like receptor signaling pathway	*Fu-Zheng*	*Radix Ginseng* (*Ren Shen*)	Ginsenoside Rh3 Protopanaxadiol	4.9 × 10^−5^ 4.9 × 10^−5^	23 15
	*Qu-Xie*	*Fructus Bruceae* (*Ya Dan Zi*)	Bruceantin Bruceine D	1.6 × 10^−3^ 3.0 × 10^−4^	7 14

## Supplementary Materials

The following are available online at http://www.mdpi.com/2072-6694/10/11/461/s1. Table S1: The basic information of the herbs applied in the research. Table S2: The resources of the compounds used in the HTS^2^ assay. Table S3: Sequences of 10 probes used in the HTS^2^ assay. Table S4: The raw experimental data of HTS^2^ assay after treatment with DMSO or TCM compounds in Figure 3 and Figure 4. The numbers are the reads of gene probes by HTS^2^ assay, which represent the abundance of genes. Figure S1: Transcriptional profile correlations of the DMSO replicates.
