# Supplementary figures and images for: Network Pharmacology to Unveil the Biological Basis of Health-Strengthening Herbal Medicine in Cancer Treatment
Source: Cancers (Basel). 2018 Nov 21;10(11):461. doi: 10.3390/cancers10110461 (PMC6266222; doi:10.3390/cancers10110461)

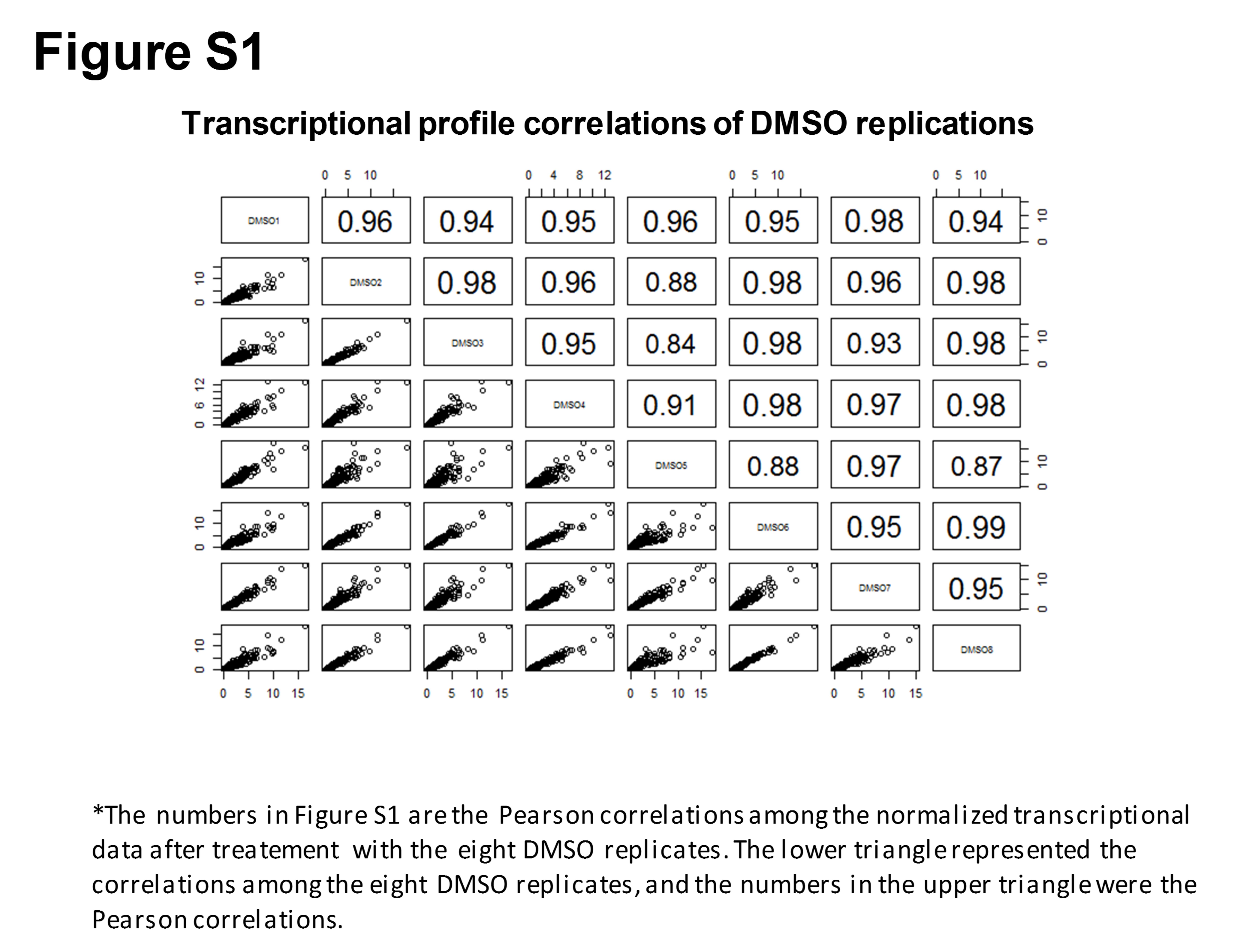

Supplement: Supplementary file 1 [file cancers-10-00461-s001.zip › revision_supplemental/FigureS1.jpg]
